# How food support improves mental health among people living with HIV: A qualitative study

**DOI:** 10.1371/journal.pone.0282857

**Published:** 2023-03-14

**Authors:** Koharu Loulou Chayama, Emiliano Lemus Hufstedler, Henry J. Whittle, Tessa M. Nápoles, Hilary Seligman, Kimberly Madsen, Edward A. Frongillo, Sheri D. Weiser, Kartika Palar

**Affiliations:** 1 Department of Global Health Sciences, University of California, San Francisco, San Francisco, California, United States of America; 2 Interdisciplinary Studies Graduate Program, University of British Columbia, Vancouver, Canada; 3 British Columbia Centre on Substance Use, Vancouver, Canada; 4 San Francisco Joint Medical Program, University of California Berkeley-University of California, Berkeley, California, United States of America; 5 Division of Psychiatry, University College London, London, United Kingdom; 6 Department of Social and Behavioral Sciences, University of California, San Francisco, San Francisco, California, United States of America; 7 Division of HIV, Department of Medicine, Infectious Diseases and Global Medicine, San Francisco General Hospital, University of California, San Francisco, San Francisco, California, United States of America; 8 Division of General Internal Medicine, Department of Medicine, San Francisco General Hospital, University of California, San Francisco, San Francisco, California, United States of America; 9 Project Open Hand, San Francisco, California, United States of America; 10 Department of Health Promotion, Education, and Behavior, University of South Carolina, Columbia, South Carolina, United States of America; 11 Center for AIDS Prevention Studies, University of California, San Francisco, San Francisco, California, United States of America; David Geffen School of Medicine at UCLA, UNITED STATES

## Abstract

**Background:**

Food insecurity is associated with poor mental health among people living with HIV (PLHIV). This qualitative study explored the mental health experiences of PLHIV participating in a medically appropriate food support program.

**Methods:**

Semi-structured interviews were conducted post-intervention (n = 34). Interview topics included changes, or lack thereof, in mental health and reasons for changes. Interviews were audio-recorded, transcribed, and double-coded. Salient themes were identified using an inductive-deductive method.

**Results:**

Positive changes in mental health self-reported by PLHIV included improved mood and reduced stress, worry, and anxiety. Participants attributed these changes to: 1) increased access to sufficient and nutritious foods, 2) increased social support, 3) reduced financial hardship, 4) increased sense of control and self-esteem, and 5) reduced functional barriers to eating.

**Conclusions:**

Medically appropriate food support may improve mental health for some PLHIV. Further work is needed to understand and prevent possible adverse consequences on mental health after programs end.

## 1. Introduction

In the era of antiretroviral therapy (ART), there is a growing number of people living with HIV (PLHIV) as a chronic condition [1, 2]. A disproportionate number of PLHIV suffer from poor mental health compared to people not living with HIV [3, 4]. A meta-analysis found that depression is nearly two times more prevalent among PLHIV than those living without HIV [4]. Further, anxiety is experienced more commonly by PLHIV (22.9%) than the general population (18.1%) [5, 6]. Depression and anxiety have been linked to lower adherence to ART and greater disease progression [7–9]. Moreover, mental health issues among PLHIV have been associated with substance use and sexual behaviors that can place individuals with suboptimal ART adherence and unsuppressed HIV viral load at high risk of secondary HIV transmission [10–14]. To address the individual and public health impacts of poor mental health, attention has been directed in recent years towards integrating mental health into HIV treatment and care [15]. Mental health interventions for PLHIV to date, however, have focused mainly on symptom reduction through biological and behavioral treatments, and reflect limited consideration of social and structural stressors such as food insecurity that increase an individual’s susceptibility to poor psychological wellbeing [15].

In the United States (US), HIV disproportionately affects structurally vulnerable groups (e.g., people of color, people who use drugs, sex workers) and subsequently contributes to their social and economic vulnerability [16, 17]. About half of PLHIV in the country are food insecure [18–20]. Food insecurity is defined as “the limited or uncertain availability of nutritionally adequate, safe foods or the inability to acquire personally acceptable foods in socially acceptable ways” [21]. Food insecurity contributes to poor mental health in both the general population and PLHIV, with studies indicating associations between food insecurity and mood, stress, anxiety, and depression [22–24]. Food insecurity may be associated with mental health status through several paths [25]. Food insecurity may create uncertainty around the ability to maintain a steady food supply or obtain sufficient food in the future, inducing stress that may lead to anxiety and depression [25]. Furthermore, food insecurity may lead people to acquire food in ways that are considered socially unacceptable (e.g., asking others for food, borrowing money for food, purchasing food on credit) [26], which in turn may generate a sense of powerlessness, shame, and guilt that are linked to depression [25, 27–29]. Food insecurity may also result in feelings of deprivation and alienation, and interfere with social relationships [25, 27, 30]. Among PLHIV, food insecurity may compound the daily stressors of HIV such as feelings of uncertainty, social isolation, stigma, and discrimination and contribute to worse overall mental health [19, 31–36]. Food insecurity and scarcity more generally may deplete individuals’ psychological resources as their cognitive bandwidth is devoted to meeting basic needs [37].

Medically appropriate food support (i.e., designed to meet medical recommendations including daily caloric and nutrient intake for populations with specific health conditions) is emerging as a potential strategy to ameliorate the negative physical and mental health impacts of food insecurity [31]. In 2014–2015, we conducted an intervention study entitled Food = Medicine that provided medically appropriate food support to PLHIV in the San Francisco Bay Area [38]. Study participants received three meals per day plus snacks, which were designed to meet their nutrition and health needs. In addition, participants received case management and enhanced nutritional counseling and education. In a quantitative analysis, we found improvements in depressive symptoms, the only mental health measure included in the outcome assessments [38]. While food support has the potential to improve the mental health of PLHIV, little is known about how food support impacts mental health. Further, what other facets of mental health besides depression may be affected by a medically appropriate food support intervention is not well understood. Greater understanding of the potential impacts of medically appropriate food support will build the evidence base required to guide policy decisions related to the provision of food and nutrition services for PLHIV in the US. Thus, we conducted a qualitative study as part of the parent Food = Medicine study to explore in-depth the broad and nuanced mental health experiences of PLHIV in the San Francisco Bay Area receiving medically appropriate food support. Our goal was to enhance our understanding of how such interventions may influence various aspects of mental health in low-income, chronically ill populations.

## 2. Methods

### 2.1. Setting and population

This qualitative study was conducted by the University of California, San Francisco (UCSF) in collaboration with Project Open Hand (POH). POH is a community-based organization in the San Francisco Bay Area that provides free meals and groceries to chronically ill clients. This qualitative sub-study was conducted as part of a parent pilot intervention study, Food = Medicine, which investigated changes in physical and mental health among POH clients with HIV and/or type 2 diabetes mellitus (T2DM) before and after receiving medically appropriate food support [38]. The food support consisted of three medically appropriate meals per day every day (i.e., twenty-one meals per week) plus snacks, based on the Mediterranean diet. Participants picked up their food twice per week at designated times from POH facilities in San Francisco and Oakland. A surrogate picked up the food on behalf of participants who were unable to pick up during these times. The parent Food = Medicine study population was comprised of adults (age 18 or older) living with HIV and/or T2DM who were current POH clients (or in the process of becoming clients), English- or Spanish-speaking, and low-income (under ~300% federal poverty line, i.e., under $35,310 for a family of one and $72,750 for a family of four) [39]. If the individuals had been a client for at least six months prior to study enrollment, they were also required to have a history of good adherence to POH services (defined as picking up at least 75% of meals or groceries in the past six months). While there are plans to expand the intervention to clients requiring home-delivered meals or a special diet (e.g., renal, vegetarian, vegan), these groups were excluded from the study to simplify procedures in the pilot phase. A full description of the intervention and main results are available elsewhere [38].

### 2.2. Participants and procedures

Study procedures were approved by the Institutional Review Board at UCSF. To be eligible for the qualitative sub-study, participants had to be enrolled in the parent Food = Medicine study and living with HIV. Food = Medicine participants living with HIV who had not dropped out of the study were recruited into the qualitative sub-study at six-months.

All Food = Medicine study participants were provided with a flyer describing the qualitative sub-study by POH staff. Those who expressed interest in participating were then approached by UCSF study coordinators to schedule an interview. Participants were recruited until saturation was reached.

### 2.3. Data collection

Before starting each interview, all study participants provided written informed consent. In-depth, semi-structured interviews were conducted by masters-level UCSF researchers trained in qualitative research methods (including ELH and TMN), including one English- and Spanish-speaking bilingual researcher, at POH facilities or another private location specified by the participant post-implementation of the Food = Medicine program. Aside from the participant and interviewer(s), no one else was present during the interviews. No prior relationship existed between the participants and the interviewers, and prior to each interview, participants were assured that the interviewers were independent of POH. Interviews were administered in English and Spanish and designed to last between 60 and 90 minutes. Interviews were conducted upon completion of the intervention at six months, or at study exit. Interviews explored participants’ experiences with the Food = Medicine program, including what and how food from the intervention was used and how they understood the intervention to impact their mental and physical health. If participants described changes in mental health related to the intervention, they were asked to describe what aspects of the intervention contributed to these changes. All interviews were audio-recorded with the permission of the participants. Post-interview memos and brief fieldnotes were written by the interviewer(s) to provide context on the interview for future reference and to share with the rest of the study team. In addition, participants provided answers to a demographic questionnaire including city of residence, gender, age, race/ethnicity, highest level of education completed, and current housing status. Upon completion of each interview, participants received $20 in cash in recognition of their time. Interviews were conducted between May of 2014 and May of 2015.

### 2.4. Data analysis

Audio-recordings were transcribed verbatim, and data were imported into Dedoose, a qualitative data management software program, for coding. A team of five researchers developed a codebook during data collection that used an integrative inductive-deductive approach involving techniques and procedures based in content coding but allowing room for emerging themes [40]. Researchers prepared a preliminary list of codes and sub-codes from the interview guide and an initial review of the data. This preliminary codebook was used for the remaining interviews and modified as new concepts emerged. When new concepts emerged, they were read and discussed by the study team and new codes were created when needed. Transcripts from the 34 interviews were double-coded by two coders at pre-determined intervals (every four transcripts) and discrepancies were discussed until consensus was reached, to increase reliability of coding [41]. Coded excerpts from the interviews related to mental health were extracted, organized, and reviewed. Researchers discussed ideas about the codes and relationships among the codes, and came to consensus about recurrent, salient themes that provide insight into whether and how participants experienced the Food = Medicine program to influence their mental health.

## 3. Results

Among the 45 participants living with HIV in the parent study, 34 individuals agreed to participate in the qualitative sub-study (Table 1), of whom 21 lived in San Francisco and 13 lived in Alameda County, which includes Oakland and Berkeley. Most participants were male, aged between 46 and 65, belonged to non-white racial/ethnic groups, and had started or completed a college education, a distribution broadly reflective of the client base of PLHIV at POH and participants of the parent study. At the time of interview, 25 participants were living in an apartment or house, and six were living in single room occupancy or nightly hotel. Twenty-nine participants reported at least mild depressive symptoms, with 9 participants reporting moderate to severe depressive symptoms. All participants had an annual household income of less than $36,000. Mean and median incomes were $16,247 (standard deviation 7,151) and $14,700 (interquartile range 10,882, 22146), respectively. Most participants received disability income assistance through Supplemental Security Income (SSI) and/or Social Security Disability Insurance (SSDI).

**Table 1:** Baseline characteristics of study participants (n = 34).

	n	%
**Residence**		
San Francisco	21	62
Alameda County	13	38
Gender*		
Male	29	85
Female	5	15
Other	2	6
**Age**		
39–45	3	9
46–55	15	44
56–65	13	38
66–75	3	9
**Race/Ethnicity** *		
White	15	44
Black/African-American	14	41
Asian/Pacific Islander	1	3
Hispanic/Latino	7	21
Native American	5	15
**Highest Level of Education Completed**		
Less than high school/GED	3	9
High school/GED	5	15
Vocational/technical school	1	3
Some college	12	35
College degree or higher	13	38
**Current Housing Status**		
Apartment or house	28	82
Single room occupancy or nightly hotel	6	18
**Depressive Symptoms**		
None	5	15
Mild	20	59
Moderate	7	21
Severe	2	6

*Participants could self-identify as multiple categories.

Most participants reported positive mental health experiences because of the intervention. A few participants noted negative mental health experiences upon program completion. During the interviews, participants described various mechanisms underlying the link between the intervention and changes in mental health.

### 3.1. Positive mental health experiences

Most participants described experiencing improved and stabilized mood. Further, most participants described experiencing reduced stress, worry, and anxiety. Five major themes that link these positive mental health experiences with the Food = Medicine program emerged.

#### 3.1.1. Increased access to sufficient and nutritious foods improved mental health

Many participants described missing meals and restricting food quantities as a coping strategy for having inadequate food prior to program participation. For these participants, access to sufficient food through the Food = Medicine program increased satiety, and in turn improved mood. A male participant in his late fifties explained:

*My belly was full a lot more*. *See, I’m the type when…my belly has food in it*, *I tend to smile a lot more than when walking around and I’m hungry*.

In addition to addressing food sufficiency, the program offered nutrient-rich foods and enhanced the quality of participants’ diets. For many, eating a high-quality and varied diet led to increased energy and sense of contentment. One male participant in his early fifties noted that increasing the amount of protein, vegetables, and fruits in the diet increased his energy level and happiness:

*I’m more happy*, *a lot more happy*. *I got more energy*, *and I got more pickup*, *more gas…I’m not down*. *I’m more up…I think I got because of the protein in the food…the veggies…I’m still just happy about eating more fruit*. *Things like that just made me feel better*.

Enhanced diet also stabilized mood for a few participants. A male participant in his fifties stopped experiencing extreme mood swings and attributed this to improved diet and blood sugar levels:

*I don’t have the mood swings that I had before*. *Now*, *maybe that was the improvement to the sugar*, *or maybe that was a tribute to the diet*. *It was either up or down*, *and I was beginning to wonder if I was bipolar*… *It’s just the extreme up or down*… *And that concerned me… [Now] it’s pretty level*… *level and smooth*, *yeah*.

In addition to changes in mood, many participants identified that consistent access to nutritious food alleviated stress, worry, and anxiety. For example, one male participant in his late fifties noted that knowing the meals were available relieved stress and anxiety around accessing food:

*Knowing that the meals were there on a consistent basis and that they were well balanced…alleviated anxiety or stress with respect to…not knowing where the next meal was coming from*, *because I had that sense of security*.

For a few participants, access to nutrient-rich foods reduced stress and improved sleep quality. One male participant in his early fifties explained that knowing he had healthy snacks available eased stress and made it easier to sleep:

*Of course it took some of the stress off*. *It made for a more restful night*. *It really did because I knew I could get up and have a snack or a healthy snack*, *not just a cookie or something sugar like that… And I could have something that’s going to settle my stomach*, *so that I could really get some sleep*. *Peaceful*, *like REM sleep*. *Yeah*, *it made a difference*.

#### 3.1.2. Increased social support improved mental health

For several participants, receiving sufficient food on a consistent basis led to increased feelings of being cared for and improved mental health, particularly among participants who were homebound. A male participant in his early forties noted that knowing that someone cares for him enough to provide three meals every day had a positive impact:

*Someone cares to do this enough to bring you your food for seven days a week*, *three meals a day*. *It’s like that helps*, *too*. *So it’s like not only was this physically beneficial*, *I would say it was emotionally and mentally beneficial*.

One male participant in his late sixties living with HIV and diabetes identified that the medically appropriate meals were carefully and thoughtfully prepared and created a sense that he was receiving ample support from POH:

*Well*, *knowing that Project Open Hand had taken the time to figure out what was needed in each meal for a diabetic*, *that was really evident*, *you know*? *The planning had taken place… So I was getting all their support… There was no bad feelings that whole time of the program*, *you know*?

Several participants described how increased feelings of being cared for during the program also influenced their experiences with stress. A male participant in his early sixties described how the comfort of having someone feed him relieved stress, particularly at times when he was experiencing many other challenges in his life:

*I think it [Food = Medicine program] reduced stress*, *too*, *because of the fact*, *like I said*, *that there’s—I knew there was all this food*. *That didn’t necessarily mean I knew there was food I would love*, *but there was always food*. *And that is beyond belief helpful… Especially when you’re already dealing with so many other issues in your life*, *it really helps to have something that you—I mean*, *think of it as being cared for*, *I mean*, *truthfully… If you had somebody who always fed you*, *it would be the same thing*.

In addition to receiving social support, some participants described how the program enabled them to offer social support to others in their community, which in turn improved their mental health. Participants explained that access to more food promoted sharing of food with their social circles, including family, partners, and neighbors. The act of offering food to others led to increased feelings of calm and content. A male participant in his late fifties who received more than a sufficient amount of food from the program described that he shared his food with others and this brought him a sense of peace:

*I even had enough that*, *you know*, *I couldn’t eat it all*, *so I was*, *you know*, *sharing it with others and stuff*, *and that all helped bring*, *you know*, *peace of mind*.

#### 3.1.3. Reduced financial hardship alleviated stress

Improved food security during the program reduced stress from financial concerns. Many participants referred to the high cost of living in the San Francisco Bay Area and described the challenges of affording food. They noted that the program alleviated some of their financial hardships and the associated stress and worries. A female participant in her early fifties noted how receiving the food eased financial worries:

*Being able to not have to worry about having enough money for food is a tremendous load off my mind*.

Other participants not only reported reduced financial concerns related to food but also other basic necessities such as housing. For example, one male participant in his late forties highlighted that rent payment is one of his greatest concerns, and the program helped ease related stress:

*Well*, *actually*, *it can’t be doing anything but helping*. *Anything that alleviates any kind of stress I welcome…I mean*, *one of the big—one of the—everybody’s big worries doesn’t just have money to spend is rent and food*. *So I would say it’s probably helped a lot*.

#### 3.1.4. Increased sense of control and self-esteem improved mood

Many participants attributed improvements in mental health to enhanced feelings of control and self-esteem. Participants demonstrated knowledge of healthy eating and a desire to consume sufficient and nutritious food. Consistent access to healthy food improved their sense of control over their diets and health choices, and in turn improved their self-confidence. One male participant in his late fifties explained that consistent access to nutritious meals allowed him to access food when he wanted, which provided a feeling of control over his life and improved self-esteem. When asked how being involved in the Food = Medicine program changed his sense of control or self-esteem, if at all, he described:

*Well*, *maybe control in the sense that*, *again*, *because the meals were consistently there*, *and they were well-balanced*, *and maybe having more control of my life because I didn’t have to*, *like I said*, *worry where I was going to get the next meal*, *and that I could just access the food when I wanted to*…*with respect to self-esteem and control*, *maybe both of them the effect would have been positive*.*”*

For some, improved sense of control was attributed to the act of participating in the program and engaging in its activities as this provided them with a sense that they have a role in caring for their own health. A male participant in his late forties described that the feeling of being proactive about addressing issues in his life gave him a sense of control:

*When I’m proactive about something*, *that automatically feeds into my confidence*, *my ego in terms of my*, *you know*, *taking the steps to address whatever the issue is*. *And*, *you know*, *that gives me that sense that I’m—I’m in control*. *I love when my doctor tells me*, *‘Oh*, *everything is under control*. *You’re doing everything that you’re supposed to be doing*.*’ I love when—when she tells me that*. *And*, *so*, *that’s*, *you know*, *that’s how I felt about this [Food = Medicine program]; that I knew that I hadn’t failed at it*.

Some participants experienced positive changes in mood because of feeling involved, and in turn changes in control. One male participant in his early seventies described that participating and adhering to the program and its processes improved his mood:

*I think it’s [mood] gotten a little better because it’s—it’s kind of like the involvement in it [Food = Medicine program]*. *Make sure that I get over here on time*, *get my meals*, *and stick to what I got*. *Yeah*, *I think it helps your mood*. *You really feel that you’re doing something*.

#### 3.1.5. Reduced functional barriers to eating alleviated stress

Some participants linked physical and mental health. They described their experiences with physical health barriers to accessing food and its associated stress and how this was addressed by the Food = Medicine program. For example, improved access to sufficient ready-made meals reduced fatigue-related barriers to cooking and eating experienced by some participants because of living with HIV, and in turn reduced stress. This was exemplified by a male participant in his early sixties who described that knowing the food was available alleviated stress around cooking:

*It was a stress relief… Sometimes people with AIDS*, *they just don’t feel like cooking*. *The fatigue is very*, *very overwhelming*. *And knowing that there’s plenty of food just relieves stress*.

Among participants with limited mobility, having food readily available at home reduced stress. A male participant in his early forties who was homebound highlighted how the program alleviated stress around the logistics of accessing food:

*I can’t stress enough as to how much of a stress reliever*, *less of a burden it was to have this program*… *I didn’t have to go out and buy the food*. *It’s here for me…[people] may not realize what an impact something like this has for people that are homebound*.

### 3.2. Negative mental health experiences associated with loss of intervention

While the program resulted in overall improved mental health for our participants, a minority of participants described negative experiences around the intervention ending, even though they were still POH clients receiving scaled-down regular services. One male participant in his early forties described experiencing negative emotions because of concerns around weaning off the program. Until realizing that his usual food support plan would still be available post-intervention, he described experiencing considerable distress over the program ending, particularly with regards to the financial aspects of acquiring food in the future if left with no food support.

*And me being crazy or whatever it was*, *thinking the whole time that*, *okay*, *I’m not getting anything else after this*. *What am I going to do*?*…So I was just thinking*, *‘It’s done*. *It’s over*. *Now what do I do*?*’ I’ve been able to save up some of my food stamps because I wasn’t buying as many groceries*. *So that was a plus…I’m like*, *I’ll have that built up and can spend that money in the beginning*. *And then when we figured it out and we remembered [that regular POH services were continuing]*, *it was just like*, *oh my gosh*. *Okay*. *Sigh of relief*. *Although it still wasn’t three meals a day and all these frozen meals and my hard-boiled eggs anymore and my vegetables or fruits I mean*. *It was still like I was relieved that I was getting at least seven meals [weekly] again*.

While participants resumed their original food support of seven to fourteen medically appropriate meals per week (or equivalent groceries), this same participant described experiencing feelings of depression because of changes in diet after completing the Food = Medicine program:

*I think things would be different if the project was still continuing… When I’m eating healthy*, *I’m feeling better*. *When I’m eating fruits and vegetables and things like that*, *I do feel better*. *So if I’m not doing that now*, *maybe that’s why I’m getting a little bit depressed along with the other things*, *too*.

## 4. Discussion

The Food = Medicine program affected the mental health of low-income PLHIV, with most participants reporting that the program contributed to positive changes in their mental health. We add to the growing evidence suggesting that food support interventions improve psychological wellbeing among PLHIV and other structurally vulnerable groups who are food insecure. Our findings are consistent with data from a qualitative study conducted by Czaicki et al., which found that food support may reduce stress, worry, and depression and increase sense of peace among PLHIV in Sub-Saharan Africa [42]. Quantitative studies that examined the impact of food support programs among other chronically ill patients and low-income individuals and families in the US have also reported reduced psychological distress and corroborate our finding [43, 44]. Participants in our study attributed positive changes in mental health to the Food = Medicine program’s contributions to improved food security, increased social support, reduced financial hardship, increased sense of control and self-esteem, and reduced functional barriers to eating. Our findings are consistent with and extend the existing literature, including the published findings of quantitative improvement in depressive symptoms in the Food = Medicine study [38], by providing an inductive, emergent understanding of how medically appropriate food support improves mental health status and experiences. Moreover, this study is the first to our knowledge to explore these mental health impacts among PLHIV in a resource-rich setting. Our results can be used to develop hypotheses about the potential mental health impacts of food support, which should be examined in future intervention studies. For example, future quantitative studies should examine whether the intervention improves stress, anxiety, and other mental health outcomes beyond depression, which was the only mental health indicator measured in the pilot study. Furthermore, future work should examine whether improvements in mental health mediate any potential impact of the intervention on HIV outcomes (e.g., ART adherence, viral suppression).

By addressing food insecurity, the Food = Medicine program also reduced stress, worry, and anxiety related to financial, as well as functional, concerns among participants. Receiving free, healthy meals from the program reduced stress triggered by financial barriers to food security. In the San Francisco Bay Area, the burden of gentrification and displacement has fallen largely on low-income residents as their wages and social assistance have not kept pace with the high cost of living [45]. While many low-income PLHIV in the San Francisco Bay Area receive monthly disability payments and/or housing assistance through public (e.g., Housing Choice Voucher Program) and private (e.g., San Francisco AIDS Foundation) institutions [46], food spending can be a significant portion of their monthly budget and compete with other demands, including rent costs. As such, minimizing their spending on meals also helped to temporarily alleviate other financial concerns around basic necessities, particularly housing. In addition to financial constraints, the intervention reduced stress related to functional limitations that led to negative psychological outcomes prior to program participation [47]. Many expressed difficulties shopping for food and preparing healthy meals due to physical conditions such as mobility impairments and fatigue, which are common among PLHIV [48, 49]. Mobility impairments and fatigue in PLHIV may be related to HIV or comorbid conditions, which have been increasing among PLHIV as the population ages [48, 49]. Our findings shed light on how food support can promote mental health by addressing financial and functional barriers to food security.

The shifts in personal control may be particularly crucial among PLHIV as living with a chronic illness may create feelings of uncertainty and loss of control [50]. Among PLHIV and those living with other chronic illnesses, feelings of personal control in relation to aspects of their daily lives enables them to cope with their disease with less psychological distress [50]. To our knowledge, our study is the first to suggest that food support–and specifically improved quantity, quality, and consistency of food supply–can increase sense of control and enable positive changes in mental health among PLHIV.

Social support was also fostered through the program and helped enhance mood and reduce stress for our participants. The link between social isolation and reduced psychological wellbeing has been well established [51]. PLHIV are particularly vulnerable to social isolation due to HIV-related stigma and disclosure challenges [52]. Low social support has been associated with depression, stress, and anxiety among PLHIV [53–55]. Previous literature suggests that social support has positive psychological impacts, including increased sense of purpose, belonging, security, and self-worth [51]. The Food = Medicine program increased social support and sense of “being cared for” by providing ample and thoughtfully prepared meals, and in turn improved mental health. Moreover, the program provided an avenue for social engagement, both at POH’s Grocery Center and in participants’ communities. We found that not only receiving but also giving social support helped to enhance participants’ mental health. Sharing food and feeding their social networks were commonly reported ways through which the program improved their mood. This is consistent with previous work that sharing program-provided food with neighbors allowed fuller participation in the community life [56], and suggests that the act of giving social support is a protective factor for psychological wellbeing [57]. We extend current knowledge by identifying that medically appropriate food support can improve mental health by enabling the means for individuals to both receive and provide social support. This finding is particularly significant given that previous research in San Francisco has found that mutual social support among food insecure individuals living in the city is often challenging and limited [58]. Food support may therefore have added benefits in such urban settings. Although food sharing in the context of individualized food support may run the risk of being framed by implementers and evaluators as a negative outcome (i.e., by diluting dietary effects on the individual), our findings indicate that it is important for facilitating the positive mental health impacts of food support.

While participants had positive experiences with the Food = Medicine program overall, a few participants reported negative mental health experiences with the termination of the intervention. Earlier work has identified that the degree of livelihood security and socioeconomic status affect whether or not PLHIV are able to transition off food support without adverse consequences to their health [59]. While there was a transition plan in place for participants to continue receiving food support upon completion of the study, our findings nonetheless provide insight into the negative mental health consequences related to reducing food support. This warrants additional attention if such interventions are to have durable, positive health impacts. Further investigation through randomized controlled trials should follow participants after transition to lower levels of food support and examine whether intervention benefits on mental health are sustained post-intervention.

Our study offers findings relevant to policy on food support for PLHIV. Given evidence of the positive health impacts of food support in this study and other studies [38, 42–44], we recommend that public funding for food support should be protected and expanded for PLHIV. Further, food support programs serving PLHIV should be nutritious and medically appropriate to deliver optimal impact on mental health. Finally, adequate quantities of food support for people at highest need should be ensured to support the mental health of PLHIV. This may mean making food available by prescription for people with health needs.

Although we sought to interview PLHIV with a broad demographic spectrum, our interviews were limited to English- or Spanish-speaking individuals who were current POH clients. This approach may have filtered out individuals who are particularly marginalized from mainstream systems of care, such as new immigrants or individuals who are experiencing homelessness. Further, we recruited clients with a history of good compliance to POH services and may have excluded individuals with particularly challenging or de-stabilizing life circumstances, such as those with serious psychiatric disorders or substance use issues. Additionally, as participation in our study was limited to individuals who remained enrolled in the study, the experiences of individuals who dropped out of the study were not captured in our findings. Due to the voluntary nature of participation, our sample may have also incurred a selection bias in which individuals who have a stronger opinion about the program may have been more likely to participate in the interviews. In particular, there is a possibility that participants shared relatively more positive experiences in hopes to influencing the chances of program continuation. To address the possibility of these sources of bias upfront, we implemented several strategies including having POH continue to provide services to participants after study end, along with designing the interview guide and training interviewers carefully to promote honest feedback including negative or neutral experiences in the program. Finally, data for this study was collected 7 to 8 years ago, potentially limiting applicability to a contemporary population of PLHIV. Nevertheless, this study offers in-depth insights into the ways in which medically appropriate food support impacts mental health among a population underrepresented in literature.

## 5. Conclusions

Medically appropriate food support such as the Food = Medicine program can address some of the multiple social and economic constraints that affect the daily lives of PLHIV and may be an effective means of improving their mental health. Further research is needed to understand and prevent possible negative consequences associated with the loss of short-term food support interventions. Research is also needed to estimate the nutritional benefits of such food support interventions. Nonetheless, our work amplifies the importance and potential of strategies that look beyond traditional healthcare to address the social and structural context of the lives of PLHIV to enhance their mental health. Federal policy concerned with the psychological wellbeing of PLHIV should protect funding for food support as part of comprehensive treatment and care. Program and policy efforts at the local, state, and national levels should continue to ensure that food support is designed and structured in a way to promote both physical and mental health.
